# An interactive web-based application for Comprehensive Analysis of RNAi-screen Data

**DOI:** 10.1038/ncomms10578

**Published:** 2016-02-23

**Authors:** Bhaskar Dutta, Alaleh Azhir, Louis-Henri Merino, Yongjian Guo, Swetha Revanur, Piyush B. Madhamshettiwar, Ronald N. Germain, Jennifer A. Smith, Kaylene J. Simpson, Scott E. Martin, Eugen Beuhler, Iain D. C. Fraser

**Affiliations:** 1 Laboratory of Systems Biology, National Institute of Allergy and Infectious Diseases, National Institutes of Health, Bethesda, Maryland 20892, USA; 2 Victorian Centre for Functional Genomics, Peter MacCallum Cancer Centre, East Melbourne, Victoria 3002, Australia; 3 ICCB-Longwood Screening Facility, Harvard Medical School, Boston, Massachusetts 02115, USA; 4 Sir Peter MacCallum Department of Oncology, University of Melbourne, Parkville, Victoria 3002, Australia; 5 Division of Preclinical Innovation, National Center for Advancing Translational Sciences, National Institutes of Health, Bethesda, Maryland 20892, USA

## Abstract

RNAi screens are widely used in functional genomics. Although the screen data can be susceptible to a number of experimental biases, many of these can be corrected by computational analysis. For this purpose, here we have developed a web-based platform for integrated analysis and visualization of RNAi screen data named CARD (for Comprehensive Analysis of RNAi Data; available at https://card.niaid.nih.gov). CARD allows the user to seamlessly carry out sequential steps in a rigorous data analysis workflow, including normalization, off-target analysis, integration of gene expression data, optimal thresholds for hit selection and network/pathway analysis. To evaluate the utility of CARD, we describe analysis of three genome-scale siRNA screens and demonstrate: (i) a significant increase both in selection of subsequently validated hits and in rejection of false positives, (ii) an increased overlap of hits from independent screens of the same biology and (iii) insight to microRNA (miRNA) activity based on siRNA seed enrichment.

## Abstract (web summary)

Analysis of RNAi screens is a multi-step process requiring the sequential use of several unrelated resources. Here the authors generate an online resource integrating RNAi analytic tools and filters into a seamless workflow, which improves the specificity, selectivity and reproducibility of the results.

## Graphical abstract

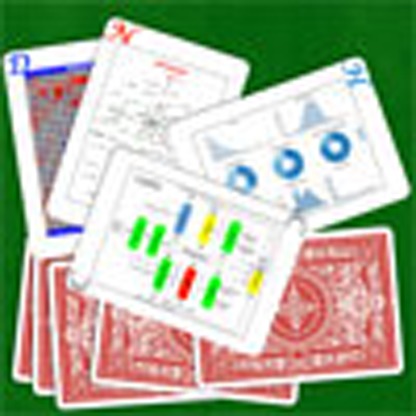

Although RNA interference (RNAi) screening technology has improved significantly in last decade, it still suffers from several limitations such as low validation rates of hits in secondary screens^1^ and limited overlap between independent screens^2^,^3^,^4^. The source of these limitations can be due to several experimental factors such as variable assay signal-to-noise, short hairpin RNA (siRNA) seed-based off-target effects and insufficient target gene knockdown^5^,^6^. Computational analysis of the screen data can identify and often correct for some of these experimental biases and improve the hit selection process, and several algorithms have been developed to reduce variation between screen plates^7^,^8^, detect off-target effects^9^,^10^,^11^, identify optimal thresholds for hit selection^12^ and to relate screen hits to specific biological processes^13^,^14^,^15^,^16^. Although some of these existing algorithms are available as isolated software tools, they are written in different programming languages and often require proficiency in computer programming for implementation. Moreover, each of these programmes have different file structures necessitating manual formatting of large data files, and they also lack data visualization features essential for effectively representing large and complex data in an intuitive manner. Hence, there is a pressing need for an integrated software platform for comprehensive analysis and interpretation of RNAi screening data.

Here we describe an integrated web-based application (available at https://card.niaid.nih.gov) for Comprehensive Analysis of RNAi-screen Data (CARD). CARD provides, within a single framework, an intuitive workflow for analysis of RNAi screen data encompassing: data upload, normalization and pre-processing, off-target analysis using multiple algorithms, regulatory miRNA prediction based on RNAi seed-based analysis, filtering based on target gene expression, integration of network resources to minimize both false-positive and false-negative hits and pathway/gene ontology (GO) term enrichment. To demonstrate the functionality of CARD, we analyse three published siRNA screens^17^,^18^,^19^. Implementation of CARD analysis on these genome-scale RNAi data sets leads to substantial improvements in hit selection, with preferential selection of hits showing higher validation rates and removal of a high proportion of genes that fail to validate. We also apply CARD to two screens that were run with different siRNA libraries but used the same biological assay^19^, and we show that CARD analysis increases the gene overlap between these screens several fold. CARD thus addresses two major challenges in the high-throughput screening field, low validation rates of primary screen hits and limited concordance between comparable screens. We also demonstrate the use of siRNA seed enrichment in CARD to provide novel biological insight to potential miRNA regulation of the screened biological processes in several of the analysed data sets. The CARD software is free for scientific and non-profit use and can also serve as a platform for securely sharing screen analyses results.

## Results

### Overview of the CARD data analysis workflow

**Figure 1:**
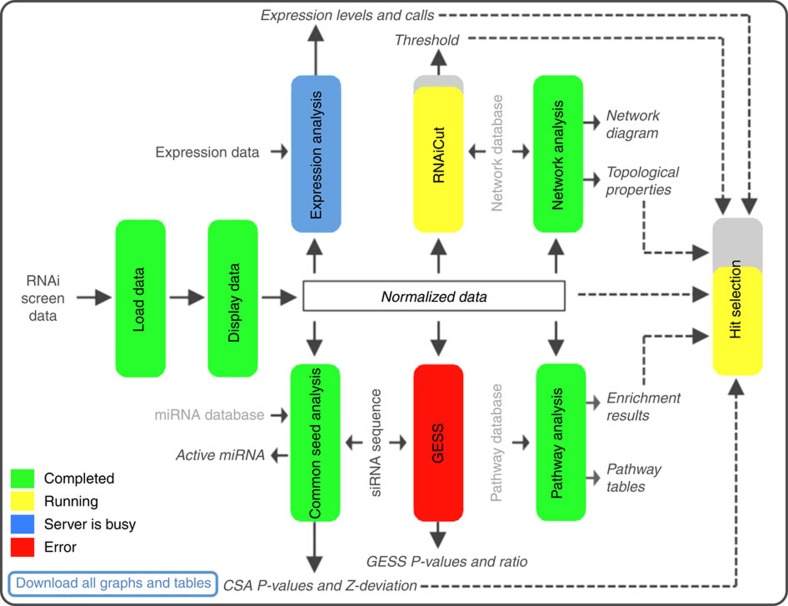
CARD project control panel. Displays the summary and status of the data analysis pipeline. Different algorithms involved in CARD data analysis are represented as coloured vertical boxes, which can be clicked to access the corresponding analysis page. The colours of the boxes indicate job status. If a job is running, progress is further represented by the coloured fraction of the box. Additional labelling describes data sets related to the screen data, biological knowledge bases used and results obtained from each of the analysis steps. Black or grey font colours represent the data specifically related to the screen or derived from general knowledge bases, respectively. Dashed lines indicate the results from individual analysis used for final hit selection.

The CARD application can be accessed at https://card.niaid.nih.gov. CARD allows users to apply all of the major steps involved in analysis of RNAi screening data within a single framework and through a user-friendly web interface. CARD not only implements many existing algorithms, but also improves them where appropriate and incorporates novel functionalities (Table 1). Importantly, the intuitive web-interface of CARD enables users without programming experience to run and monitor the progress of multiple sophisticated algorithms from an interactive control panel (Fig. 1). As the screen results and their interpretations are dependent on a multitude of factors (including biological system, assay type, experimental conditions, controls and so on), CARD provides users the flexibility to conveniently customize the analysis parameters. CARD also uses cutting-edge data visualization techniques that allow users to interact dynamically with the figures and tables displayed on the web-browser to facilitate the hit selection process. Results generated in any CARD project can be stored and securely shared with collaborators via a project portal (Supplementary Fig. 1).

### CARD control panel

The control panel page summarizes the steps/algorithms involved in the CARD application and the flow of data between different steps (Fig. 1). Each vertical box represents a step of CARD, which involves the input and output of data. Mouse-over provides a brief summary of the step and the user can visit the corresponding page by clicking the box. The box colours represent the current status of the page. If a process is running, partially filled yellow represents the approximate percent progress. Once a step is successfully completed, the corresponding box turns green. As Hit Selection uses information from the previous steps, it turns green only when all previous steps have been completed. All the results associated with a specific project can be downloaded as a compressed file by clicking ‘Download All Graphs and Tables’.

### Uploading screen data in CARD

**Figure 2:**
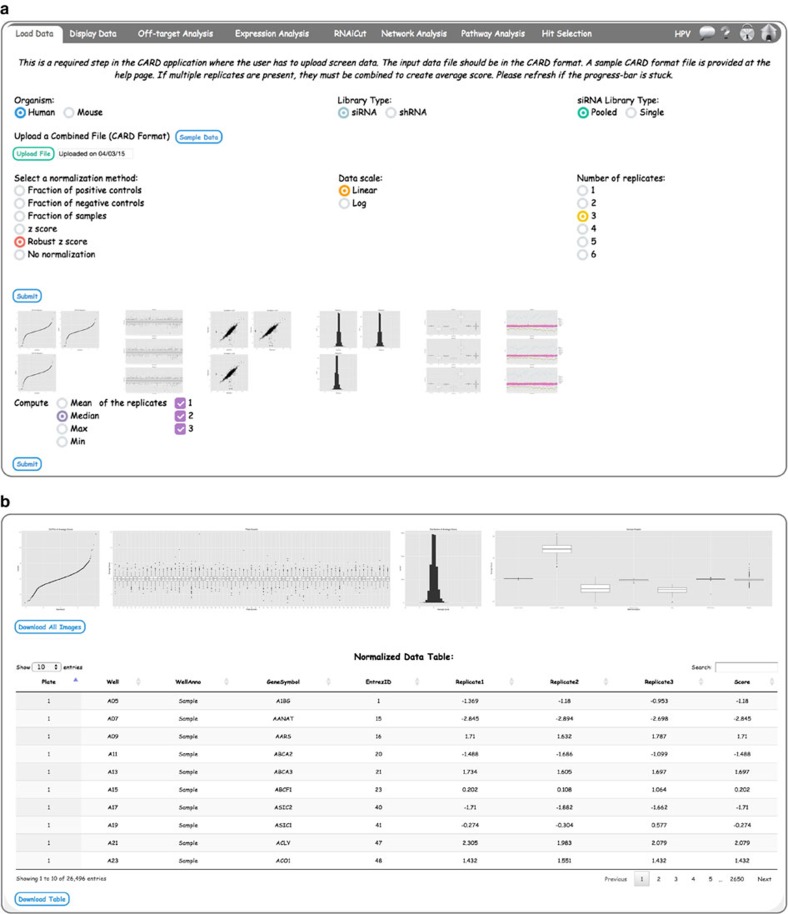
Loading and normalization of screen data in CARD. (**a**) Loading and preprocessing of screen data. If multiple replicates are present (as shown here for HPV screen data), the user can compare the replicate correlations and decide if any of the replicates should be rejected. The thumbnail images of the screen data can be clicked to enlarge the graphs (see Supplementary Figs 2 and 3 for details). (**b**) The data properties of three combined replicates are shown.

This is a required step in the CARD application where the user has to upload screen data (Fig. 2a). Sample CARD format files are provided in the help page (accessed through question mark icon at top right) and sample files for both individual and pooled siRNA screens are shown in Supplementary Tables 1 and 2 (see Input Data Formats in Methods for details). CARD allows the user to upload either raw or normalized data (‘No normalization’ option should be chosen if already normalized data is uploaded). Other input parameters to be selected on the *Load Data* page include ‘Organism’ (used for mapping organism specific databases), ‘Library Type’ (siRNA or short hairpin RNA (shRNA)), ‘siRNA Library Type’ (used in off-target prediction), ‘Select a normalization method’, ‘Data scale’ and ‘Number of replicates’ (Fig. 2a). Once the job is submitted, processing will start at the backend server and the progress bar will show the percentage progress of the job. After the processing is complete, CARD creates a number of figures indicating data properties, which are shown as thumbnails (Fig. 2a, lower panels). The user can view a larger-scale figure by clicking on any thumbnail (see Supplementary Figs 2 and 3 for details). These figures include a QQ-plot, Replicate Histogram, Replicate Boxplot, Replicate Correlation (not shown if only one replicate is available), Sample Boxplot and Jitter-plot. As pooled shRNA screen data are not associated with plates, Replicate Boxplot and Jitter-plot will not be created for shRNA screen data. If multiple replicates are present, the user can choose to eliminate any potential outlier replicate(s) by deselecting the corresponding check box(es). The user can further combine replicates from a choice of functions (mean, median, minimum or maximum) and after processing, the figures and tables for the combined replicate data will appear (Fig. 2b).

### Screen data display in CARD

**Figure 3:**
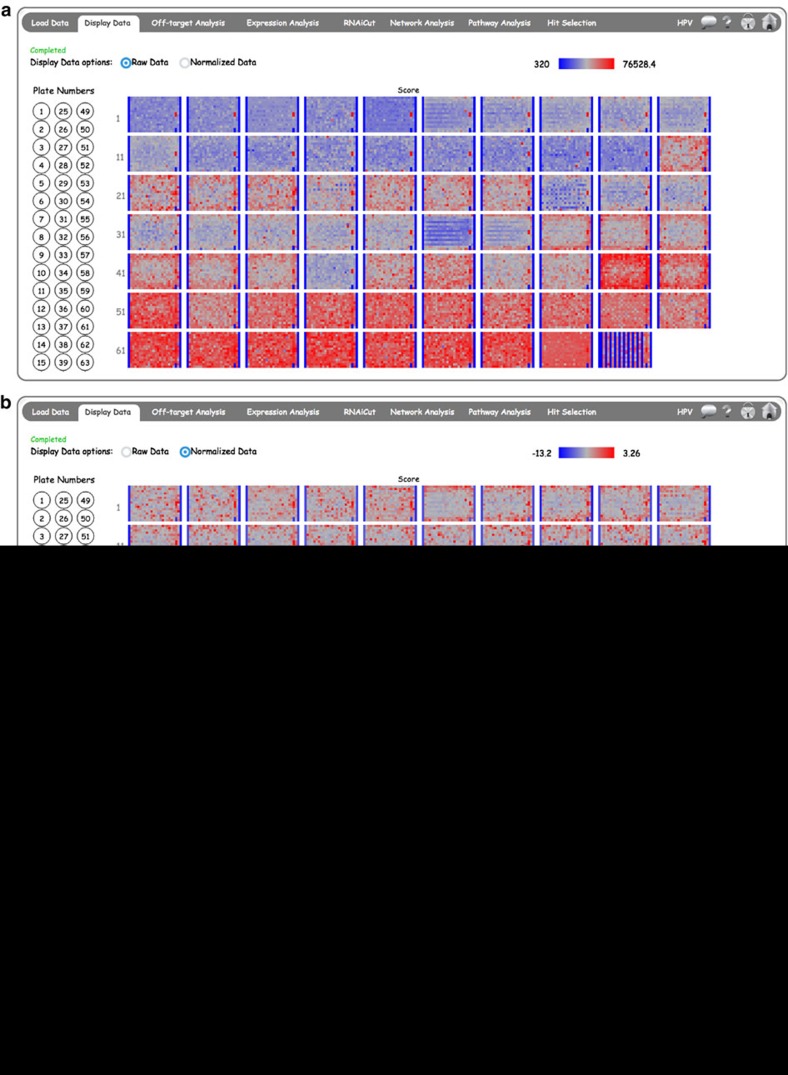
CARD *Display Data* page. An interactive display of all plates in a sample genome-wide screen is shown. The left panel circles show the plate numbers. Selecting ‘All’ will display thumbnails of all the plates. By selecting the radio button the user can compare (**a**) raw data and (**b**) normalized data. (**c**) Single-plate display with highlighting of gene-specific siRNAs in single wells. Wells can be highlighted based on annotation by selecting buttons from right panel. Black borders highlight wells annotated as ‘sample’ in the input file. Hovering on a specific well will provide siRNA- and gene-specific data.

The ability to visualize siRNA screen data in a plate format can allow the user to identify any systematic experimental bias, such as variation between screen plates and edge effects within a specific plate. Figure 3a shows an example of systematic variation between plates in the CARD *Display Data* page, where some of the plates are predominantly red or blue. We applied robust *Z*-score normalization to reduce these plate-wise variations. By clicking the ‘Normalized Data’ radio button (Fig. 3b), the user can visualize the normalized data. Data can also be visualized in single-screen plates by clicking specific plate numbers from the left panel (Fig. 3c). In this single-plate mode, data from a specific plate are visualized on the right and data from all plates are displayed as a ‘waterfall/snake plot’ on the left. The user can mouse-over on each of these plots to obtain detailed information on individual wells, such as Gene Name and ID, Well Annotation, Well Number, *Z*-score and so on. This information is shown for a specific well (marked by the black arrow) in Fig. 3c. The red line in the snake plot shows the relative position of the well in the overall population, allowing the user to compare the scores in a specific plate to the overall population. Different types of well annotation (based on the user-defined labels included in the ‘WellAnno’ column as shown in Supplementary Tables 1 and 2) can be highlighted by selecting the buttons from the right panel. If multiple replicates are present, the average of the replicate plates will appear on the Display Data page. The colour scale is automatically adjusted to the dynamic range of the values.

### Application of common seed analysis (CSA) in CARD

**Figure 4:**
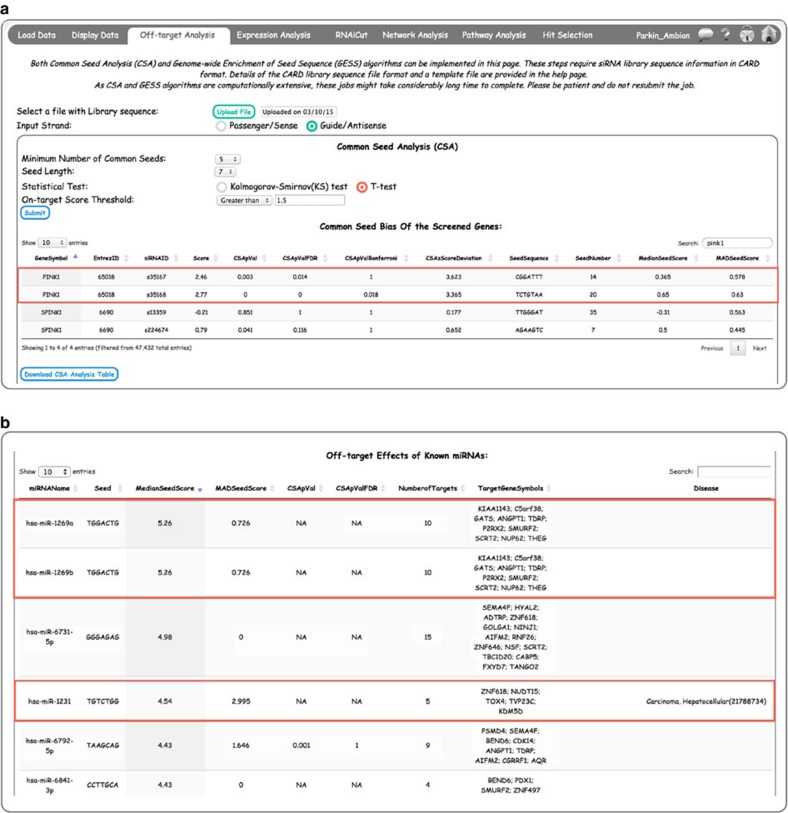
CARD *Off-target Analysis* page: CSA and miRNA prediction. (**a**) Common seed analysis (CSA) results show seed biases of individual siRNAs. Note that two siRNAs targeting PINK1 (a strong hit in the Parkin screen) show seed bias with a CSA pVal close to 0. However, CARD-calculated *Z*-score deviation of >3 identifies both siRNAs as true hits and rescues them from CSA filtering. (**b**) Matching of screen siRNA seeds to known miRNAs to predict putative miRNAs regulating the biological process analysed in the screen. Note highlighted seeds with high median seed scores in the HDAC inhibitor siRNA screen match to specific miRNAs, which also scored positive in the HDAC inhibitor miRNA screen (see Methods for details).

If a set of siRNAs sharing a common seed sequence, but not necessarily designed to target the same gene, show a significantly stronger phenotype compared with the rest of the screened siRNAs, it is suggestive of a seed-based off-target effect. The *Off-target Analysis* page provides CSA^9^ (Fig. 4a) and Genome-wide Enrichment of Seed Sequence (GESS^10^; Supplementary Fig. 4) algorithms for identifying off-target siRNAs and off-targeted genes, respectively. Both of these algorithms use siRNA sequence information, hence, a file containing EntrezID, siRNAID and Sequence should be uploaded in this page. An example of the format for an siRNA sequence file is shown in Supplementary Table 3. Sequence type should also be selected using the appropriate radio button, that is, ‘Sense’ or ‘Antisense’.

For CSA, the user has to provide other programme parameters like ‘Minimum Number of Common Seeds’, ‘Seed Length’, ‘Statistical Test’ and ‘On-target Score Threshold’ (Fig. 4a). More information about each of these parameters can be obtained by hovering on the parameter text. After selecting parameters and submitting, the results of CSA appear as two tables. The first table (Common Seed Bias of the Screened Genes) shows the CSA results for the siRNAs in the screen (Fig. 4a). The table contains information on *P*-value computed on the basis of the user-selected statistical test and corrected *P*-values (after FDR and Bonferroni corrections). The table also contains a measure of ‘CSAzScoreDeviation’. This is an extension of CSA that we introduced in CARD. Specifically, it estimates the on target effect of each siRNA after correcting for any seed-based off-target effect. This calculation can salvage some siRNAs that may have a significant off-target effect, but their on target effect is considerably greater (an example of this is shown in Fig. 4a for siRNAs against *PINK1* in the Parkin siRNA screen). The CSA zScoreDeviation is computed using equation (1)


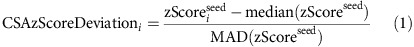


Where 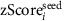 is the *Z*-score of siRNA ‘*i*’ with a certain seed sequence. MAD (zScore^seed^) and median (zScore^seed^) are the Median Absolute Deviation and median of the siRNA scores with the same seed sequence. The first CSA table also contains the seed sequence, the number of siRNAs with that seed sequence and the median and MAD of the siRNA scores with that seed sequence. The user can sort this table based on any of the columns and filter it using the search box.

As a seed-based off-target effect is caused by siRNAs functioning through an miRNA-like mechanism, comparing the active seed sequences in the screen with miRNA databases can provide insight into miRNAs that may be associated with the biology being studied. CARD provides this additional feature as part of CSA. Each row in the ‘Off-target Effects of Known miRNAs’ table is a known miRNA, and the table contains information on miRNA-name, Seed, MedianSeedScore (median of all the siRNA *Z*-scores with the seed sequence), MADSeedScore (MAD of all the siRNA *Z*-scores with the seed sequence), CSApVal, CSApValFDR and the number and list of potential target genes that contain a 3′ untranslated region (UTR) match to the miRNA seed (Fig. 4b). To provide additional validation that the seed-based miRNA-like effects in the screen are likely highlighting important biology, the genes in the TargetGeneSymbol column can also be filtered using the ‘On-target Score Threshold’ parameter (Fig. 4a). This allows the user to only display genes whose on-target siRNAs (not those that contain the enriched seed) also pass a user-defined *Z*-score threshold. This orthogonal use of both off-target and on-target effects in the screen is a powerful feature of the CARD application. The Disease column in the miRNA table (Fig. 4b) provides information on known association of the miRNAs with human diseases based on the human miRNA-associated disease database (HMDD)^20^.

### Filtering screen hits based on mRNA expression data

**Figure 5:**
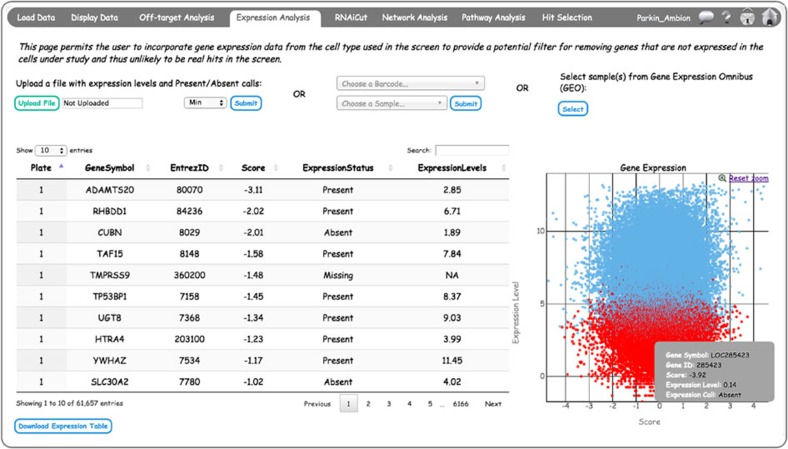
CARD *Expression Analysis* page integrates genes expression data with screen scores. Gene expression data can be uploaded from a user-provided data set, gene expression barcodes or from the GEO database. Each circle in the scatterplot represents a gene. Detailed expression information can be obtained by mouse-over (example is shown in gray box). If Present and Absent calls for the genes are included in the uploaded data set, they are shown as blue and red, respectively.

Gene expression analysis is included in the CARD application to permit data filtering based on the assumption that if a gene is not expressed in the target cells being studied, it is unlikely to be a valid hit in the RNAi screen. The user can choose from three different options for expression filtering (Fig. 5). If the user has gene expression data (either microarray or RNA-seq) from the screen cell type available, it can be uploaded using the ‘Upload a file with expression levels and Present/Absent calls:’ option. Details of this input file format are provided in Supplementary Table 4. The second option is to choose expression calls from the Gene Expression Barcode database^21^. This database contains information on whether a gene is expressed or not, based on the barcode algorithm from expression distribution of the probe over a large number of samples. The algorithm has been applied to three human and two mouse Affymetrix platforms for which a large amount of expression data is available over a range of conditions. The user first has to select a platform from the top drop-down box and select the most relevant sample from the bottom drop-down box. Both of the drop-down boxes have an auto-completion feature using ajax technology. There is a third option where the user can use any gene expression data set submitted to the GEO database (http://www.ncbi.nlm.nih.gov/geo/). This is useful when the user did not generate expression data under the screen conditions, but can identify a relevant gene expression data set, perhaps from the same cell line, in the GEO database. If this option is selected, a pop-up window will appear where the user can provide GSM number(s) of the gene expression data sets from GEO. If the user provides multiple GSM numbers, they should come from the same array platform and they are averaged in CARD based on the option selected in the drop-down box (minimum, maximum or median).

The expression scatterplot shows screen scores versus expression levels (Fig. 5; bottom right). Similar to previous scatterplots, this figure is also interactive; the user can drag, zoom and hover on the points in the graph to get detailed information. The table shows the expression levels and expression status, and if the expression data of a screened gene is not available, its expression level and status are marked as NA and Missing, respectively. If Present/Absent calls are not available in an expression data set, all the genes present in the expression data will be marked as Present by default.

### Identifying hit selection thresholds using *RNAiCut*

**Figure 6:**
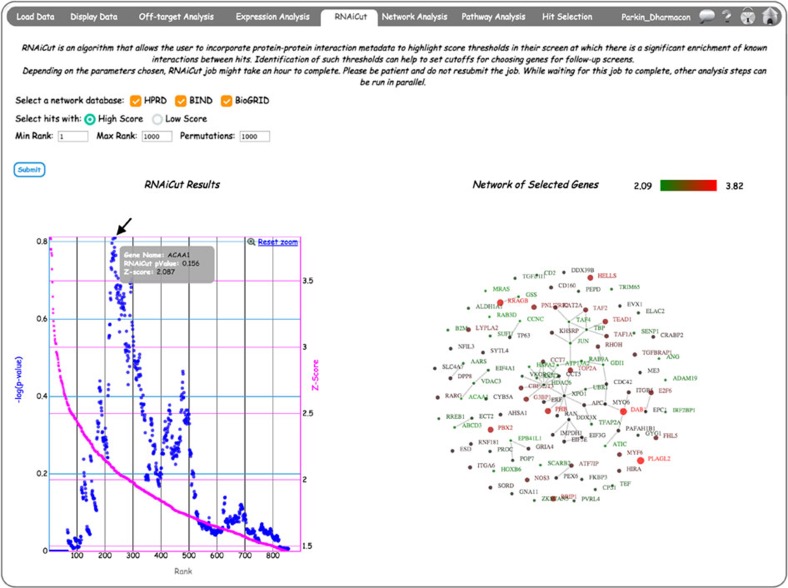
Implementation of the *RNAiCut* algorithm in CARD. Interactive representation of sample results from RNAiCut analysis (see Methods for details). The scatterplot shows –log(*P*-value) and *Z*-score as function of gene rank (sorted based on the screen score). By mouse-over on any of these scatter plots, details like Gene Symbol, RNAiCut *P*-value and *Z*-score will appear. The maximum –log(*P*-value) (denoted by arrow), occurs around *Z*-score 2.087. Clicking on this point renders an interaction network on the right among genes with *Z*-scores greater than 2.087.

*RNAiCut* attempts to identify a biologically meaningful threshold for hit selection by combining screen scores with protein–protein interaction (PPI) network data^12^. Specifically, it identifies the probability that the connectivity of the hit genes, identified using a specific threshold, are higher than what is expected by random chance. For single siRNA screens, the *Z*-scores of different siRNAs targeting the same gene are averaged to rank the genes (Fig. 6). The user can select one or multiple network databases by checking the appropriate boxes. If multiple networks are selected for the analysis, the union of the networks is used. The user can choose the directionality of the analysis, providing flexibility to run it on either positive or negative scoring genes. In the Min Rank and Max Rank textboxes, the user has to provide the gene rank range for running RNAiCut, these values should be integer numbers. The user can also choose the number of permutations to be used for estimating the RNAiCut *P*-value. RNAiCut uses a non-parametric method of estimating significance/*P*-value by shuffling the network. In the CARD implementations, the network shuffle is carried out such that node degrees are conserved. The higher the permutation number, the more accurate the *P*-value estimation; however, this increased accuracy requires greater analysis time.

Once the RNAiCut analysis is complete, a scatterplot appears showing both siRNA screen *Z*-score and –log(*P*-value) of interactions between hits as a function of rank on two different axes (Fig. 6). The premise of RNAiCut is that at any chosen point along the gene rank distribution, the *P*-value represents the frequency of known interactions among genes with screen scores stronger or equal to that at the chosen point. Based on the RNAiCut algorithm, the minimum *P*-value for likely interactions (which equates to the maximum –log[*P*-value] in the graph) should correspond to the most biologically meaningful threshold. If there are multiple local maxima for –log[*P*-value], the user should take into account both *P*-value and screen *Z*-score to decide the optimal threshold. The scatterplot is interactive, hence, the user can click on any of the points of the scatterplot, and a network will appear on the right between the genes that have scores exceeding the chosen point. The colours and the sizes of a node depend on the screen score. Figure 6 shows an example where the –log[*P*-value] distribution was clicked at the maximum point (Gene: *ACAA1*, *Z*-score: 2.087), and the rendered interaction network shows all genes with *Z*-scores ≥2.087. Note that the rendered Network of Selected Genes is also interactive; allowing the user to click and move connected networks to facilitate data mining. Only genes present in the chosen network databases will be used for this analysis. Hence, the scatterplot *x* axis corresponds to the Rank among genes that are present in the network database (it will always be less than or equal to the Max value chosen by the user).

### Network analysis of screen hits in CARD

**Figure 7:**
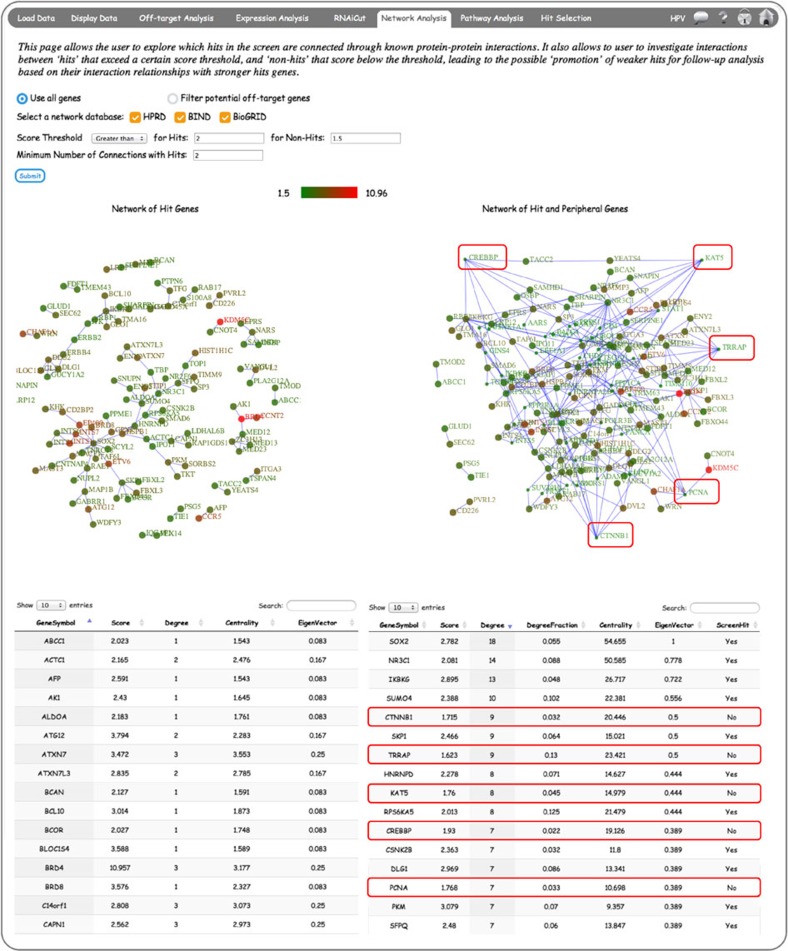
CARD *Network Analysis* integrates protein–protein interaction (PPI) data with screen data. The left panel shows screen hits defined by chosen score thresholds that are connected based on the selected network databases. The right panel includes both ‘hits’ and ‘non-hits’ (lower scoring genes from a user-defined threshold) that are connected based on PPI network data. The hit genes in this network are represented by larger circles, and circles are coloured based on the screen score. The red boxes highlight examples of ‘non-hits’ with multiple ‘hit’ interactions from the HPV screen data set (Supplementary Table 7).

The network analysis in CARD serves two purposes. First, by mapping genes onto PPI networks to identify connections between hit candidates, it highlights higher confidence hits for follow-up analysis. This strategy can potentially reduce false-positive rates with the assumption that if a putative hit is connected with other hit genes, it is more likely to be a real hit. Second, it attempts to identify false-negative candidates by promoting lower scoring ‘Non-Hits’ (weak hits) through their known interaction with stronger hits in the screen. The user has the option to filter the potentially off-targeted genes before running *Network Analysis* (Fig. 7). If this option is selected, the user has to provide the *P*-value and *Z*-score deviation thresholds to be used for removing off-targeted genes. The user then chooses the screen *Z*-score directionality and cutoff for hits and non-hits, and also the network databases (HPRD^22^/BIND^23^/BioGRID^24^) that they want to use. If multiple network databases are selected, the union of the networks will be used to run this analysis. The results are shown as interactive tables and figures, with the network of hit genes only on the left, and the more extensive network of both hits and peripheral-genes (weak-hits) on the right (Fig. 7). In the network rendition, bigger and smaller circles represent the hits and peripheral genes, respectively. Colours represent the screen score and their scales are chosen to represent maximum dynamic range. The same colour scale is used for both of these networks. Adjusting the input parameters, that is, score thresholds and minimum number of connections with hits for genes can alter the complexity of the network visualization.

### Pathway analysis of screen hits in CARD

In a further feature that allows the user to identify functional enrichment among the top scoring genes in their screen, CARD analyses enrichment of screen hits with canonical pathways and GO terms (*Pathways* will generally refer to both). Similar to *Network Analysis*, the user has the option to filter the potentially off-targeted genes before running *Pathway Analysis* (Supplementary Fig. 5). The user can then choose one or multiple data sets from kyoto encyclopedia of genes and genomes (KEGG)^25^, Reactome^26^ and GO^27^. CARD uses the one-sided Fisher’s exact test to identify if the hits are predominantly coming from certain pathways. CARD provides the flexibility to choose hits in positive and negative directions of *Z*-score by selecting from the drop-down menu, ‘greater than’ or ‘less than’, respectively.

Results from KEGG and Reactome databases are shown as separate tables. As GO terms are classified into three categories, that is, Biological Process, Molecular Function and Cellular Compartment, each of these are represented as separate tables. The tables contain *P*-values, corrected *P*-values, number of genes from the pathway present in the screen, number of hits from the pathway based on the criteria selected and the list of hits from the pathway. As many GO terms are either too specific or too generic, users can filter them out from the list to come up with a more informative list. Each of the tables can be filtered based on the pathway size and sorted based on any of the columns. The user can search for a specific gene by typing the gene name in the search textbox. Each of these tables can be downloaded as a .csv file by clicking download the button. Supplementary Fig. 5 shows an example table from the KEGG pathway database.

### Integration of multiple analysis filters for Hit Selection

**Figure 8:**
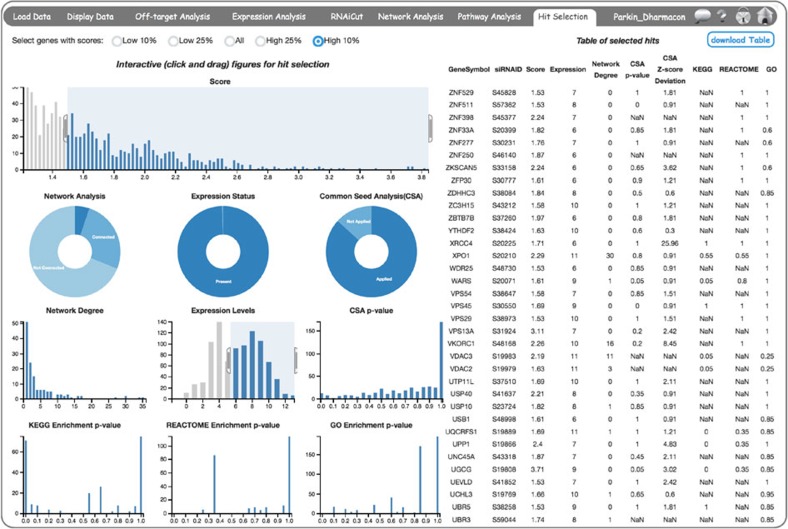
CARD *Hit Selection* page. Hit Selection page providing both a summary and details of the hit selection process. Choosing a fraction of the genes (10%) from the top radio buttons expedites data loading from genome-scale screens. On the left side, a summary of the results is shown as interactive bar and pie charts. On the top row, a bar chart shows the histogram of RNAi screen scores. The Network Analysis pie chart shows fraction of genes connected, not connected and unknown as obtained from the Network Analysis page, whereas the Network Degree histogram shows the degree distribution of the connected genes. Similarly, the gene Expression Status pie chart shows the fraction of present/absent genes and the Expression Levels histogram shows an often-bimodal distribution of expression, where the left mode corresponds to likely absent genes. The common seed analysis (CSA) pie chart shows the fraction of siRNAs for which CSA was applied and not applied, then the CSA *P*-value distribution is shown specifically for the applied subset. As KEGG, Reactome and GO databases have information for only a subset of the genome, the histograms show the enrichment *P*-values of the selected genes present in those databases. Mouse-over on chart headers provides description of the chart. On the right panel, an output table of the selected hits is provided. Each of the charts is interactive, such that clicking and dragging to select a subset of genes applies filters that are dynamically reflected in the table of hit genes.

The *Hit Selection* page allows the user to incorporate the results from all the previous steps in CARD analyses to identify an optimal set of genes for follow-up studies (Fig. 8). The left panel shows a summary of the screen results as histograms and pie charts, whereas the right panel shows a table of candidate hit genes with screen data values. The user can scroll down the table, which is sorted based on the gene symbol. If a process is not completed, the corresponding column in the table will have NaN values. As many genes are not represented in the KEGG, GO and Reactome databases, these genes will have NaN values in the Hit Selection page even after Pathway Analysis is complete. The panels on the left side of the Hit Selection page are interactive and interwoven. Hence, the user can select a subset of siRNAs by brushing the histograms or clicking on a specific slice of the pie charts, and all figures and the table will be redrawn based on the siRNAs selected or the filter applied. The user can apply a combination of filters from two or more figures. Clicking the ‘download Table’ button will download the filtered list of results. By checking radio buttons on the top of the page, users can specifically focus on either the top or bottom of the score distributions from where hits are usually selected.

### CARD analysis preferentially identifies validated screen hits

**Figure 9:**
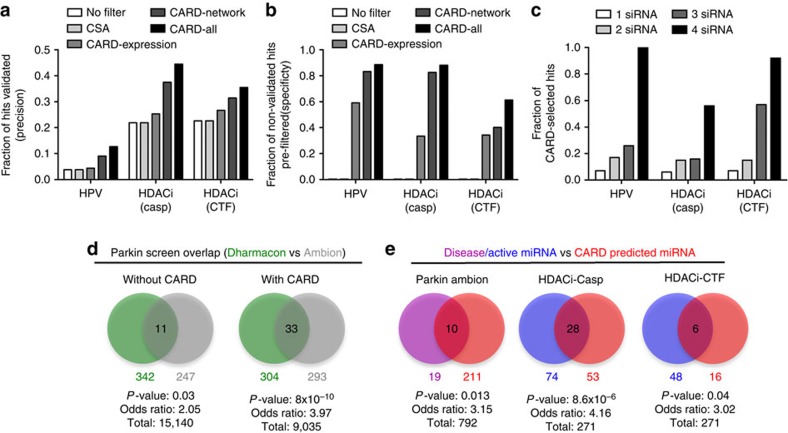
CARD analysis of genome-scale siRNA screens leads to improved hit selection and prediction of active miRNAs. (**a**) Comparison of Precision (fraction of the hits from primary screen that are validated in the secondary screen) and (**b**) Specificity (fraction of non-validated hits in the secondary screen that would be eliminated by CARD filters in primary screen) with either no filter, individual filters and all filters using CARD. Both precision and specificity increase when CARD filters are applied and are highest with all filters combined. (**c**) Fraction of hits selected by applying all CARD filters among genes validating with different numbers of single siRNAs in the analysed HDAC inhibitor (HDACi-Casp and HDACi-CTF) and HPV screens. Additional details of parameters used in the applied CARD algorithms are provided in the Methods. (**d**) Comparison of the hit gene overlap between the Parkin Dharmacon (green) and Ambion (grey) screens with and without using CARD. The statistical significance of the overlap increases considerably after applying CARD filters. (**e**) Comparison of active miRNAs identified from miRNA mimics in the HDAC inhibitor screen (blue), or Parkinson’s Disease associated miRNAs from literature curation (purple), with predicted active miRNAs from siRNA seed enrichment in CARD analysis (red). A low *P*-value from Fisher’s exact test and an odds ratio >1 demonstrate the overlap significance.

To test the functionality of the CARD software in improving primary screen hit selection, we first analysed two genome-scale siRNA studies for which secondary validation data sets were available: a screen for sensitization of cancer cells to histone deacetylase (HDAC)-inhibitor (vorinostat)-induced cell death^17^ and a screen to identify the human genes responsible for mediating repression of human papilloma virus (HPV) oncogenes^28^. We plotted three different features of hits used in the validation experiments as a function of the number of independently validating siRNAs in the secondary screen (Supplementary Fig. 6). There is a clear trend showing that higher validation rates correlate with the gene and siRNA features (Network degree, expression level and *Z*-score deviation) that can be selected for in the CARD analysis workflow. Hence, we decided to use these features for filtering hits, as they appear to preferentially select hits with higher validation rate. For HDACi-Casp, HDACi-Cell Titre Fluor (CTF) and HPV screen data sets (see Methods), we used these features in combination (hits satisfying selection criteria from all three features) for identifying hits. Supplementary Table 5 lists these properties and the corresponding threshold values used for hit selection. To compare the hit selection rate of CARD, we chose the same *Z*-score cutoff for hit selection used in the original studies (marked by *). To then test the utility of CARD, we compared the validation rate of the hits with or without CARD filtering. We first tested the fraction of high confidence hits selected after applying individual (network, expression, CSA) and combined filters in CARD that validated in the secondary screen compared with the validation rates of the hits without filtering (Fig. 9a). We considered a hit ‘high confidence’ if three or more independent siRNAs replicated the pooled siRNA primary screen phenotype. In all three screen data sets, the validation rate is increased by twofold or more with application of combined CARD filters (Fig. 9a). We also tested if CARD preferentially rejects genes that are not validated in the secondary screens. We computed the fraction of unvalidated hits rejected by CARD (specificity; Fig. 9b). For all three data sets, the high-specificity fraction underscores the value of CARD in prioritizing higher confidence hits. In the secondary validation screens, the four siRNAs from the primary pooled screen were deconvoluted and used separately. The higher the number of these four independent siRNAs validating in the secondary screen, the greater is the likelihood that a candidate gene is a true hit. We therefore grouped the hit genes from the secondary screen based on their validation rate, and for each of these groups, we identified the fraction of hits selected by CARD (Fig. 9c). For all three data sets, CARD showed increasing selectivity for genes with higher validation rates.

### CARD analysis improves the overlap between related screens

Poor overlap of hits obtained between different independent screens has raised concerns about the reliability of RNAi screen results^2^. To determine whether the CARD analysis workflow could improve the overlap between screens evaluating the same biological process, we used a screen for genes associated with Parkinson’s disease that compared two independent genome-scale siRNA libraries: a Dharmacon library using pools of four siRNAs and an Ambion library used three siRNAs per gene screened individually^19^. We applied CARD filters to both data sets with the parameters listed in Supplementary Table 6. Using only the *Z*-score thresholds used in the original study, we identified 342 and 247 hit genes whose knockdown increase mitochondrial translocation of Parkin, and found 11 overlapping hits between these two sets (Fig. 9d). We applied CARD analyses to these data sets to first identify a set of core hits that passed gene expression, network and CSA filters with the thresholds provided in Supplementary Table 6. We then used *Network Analysis* of CARD to identify additional peripheral hits connected to the core hits (see *Network Analysis* section for details) from both data sets. We only selected the subset of peripheral hits that also passed the expression and CSA filters. Finally, we combined the peripheral and core hits to create a final set of CARD hits for both of these data sets. After applying these analyses in CARD, we found that the number of overlapping genes increased almost threefold (Fig. 9d). To test whether this overlap is statistically significant, we applied Fisher’s exact test with a modified number of background genes, considering only the subset of screened genes expressed and present in the network databases. The extremely low *P*-value of 8 × 10^−10^ (compared with 0.03 before CARD analysis) indicates that the statistical significance of the overlap has increased significantly. This analysis also implies high false-negative rates can be responsible for poor overlap between data sets and *Network Analysis* can help to address this issue.

### Identification of active miRNAs using CARD

As described earlier, CSA identifies seed sequences in an RNAi screen with enriched phenotypic effects compared with background. The siRNAs with these strong seed biases cause off-target effects through miRNA-based mechanisms^9^,^10^,^29^. We first used CARD to predict active miRNAs in the Parkin Ambion screen from the siRNAs that showed strong seed bias. When we compared them with human miRNAs known to be associated with Parkinson’s Disease, we found significant statistical enrichment (Fig. 9e). Similarly, we tested the miRNA predictions from CARD analysis of the HDACi-Casp and HDACi-CTF siRNA screens by comparing the miRNA mimic screens that were run using the same assays. Specifically, we categorized miRNAs with median seed scores (from the siRNAs with the same seed sequence) greater than 1 as hits for the HDACi-Casp screen and less than 1 as hits for the HDACi-CTF screen. The same cutoffs were also used in the miRNA mimic screen to identify miRNA hits. The numbers from each of these analyses and their overlaps are shown in Fig. 9e. For both screens, overlaps were greater than what would be expected by random chance based on one-sided Fisher’s exact test, particularly for the HDACi-Casp screens. These analyses suggest that active miRNAs identified from CARD analysis of siRNA screen data could highlight important biology for further investigation and validation.

## Discussion

We have described the development and application of CARD, a novel software application for comprehensive analysis of RNAi screen data. Through the re-analysis of published genome-scale siRNA screens, we have demonstrated that CARD filtering of primary screen data can preferentially select for hits that are more likely to validate in secondary screens, can reliably filter out likely false-positive hits, and can improve the hit overlap rate of screens addressing similar biology. We also demonstrate that by taking advantage of the off-target effects of siRNAs acting through miRNA-like mechanisms, we can use such siRNA ‘seed enrichment’ to implicate known miRNAs, which might regulate the biological process being studied.

CARD uses a client-server software architecture, which implements interactive data visualization on the client side and robust statistical analyses on the server side. The modular architecture of the CARD application will allow us to incorporate additional novel analysis algorithms into future versions of the software. For example, CSA is more complicated for shRNA-based screens because of heterogeneous seeds generated from the same shRNA, however, once off-target analysis algorithms for shRNA-based screens become more mature, we plan to incorporate them within the CARD framework, which will extend the utility of the software for shRNA screen data analysis. Similarly, many of the existing steps in CARD could also be applicable to the analysis of screen using CRISPR/Cas9 genome editing^30^ or CRISPRi transcriptional repression^31^, which will allow the development of a comparable analysis pipeline, but again this will require a clearer understanding of the frequency and nature of off-target effects using this alternative technology.

An additional challenge in RNAi-based screens is the comparison and integration of both primary and secondary screen data into a single data analysis workflow. Although we plan to address this in future versions of CARD, we would point out that secondary screen data can be readily analysed as a separate screen data set in the existing version of the software. Additional areas for future development include extension to other organisms besides human and mouse and, depending on user demand, implementation of a cloud-based version to scale the software data handling as required.

## Methods

### Software architecture

CARD uses a client-server model that enables users to employ a web browser interface to create and share projects, select programme parameters and upload screen and sequence data to execute computational analysis algorithms. The submitted jobs are executed at the back-end using R-scripts, which relies on custom function codes and uses multiple *bioconductor* libraries such as *org.Hs.eg.db*, *org.Mm.eg.db*, *GEOquery*, *limma*, *seqinr*; and CRAN packages including *ggplot2*, *igraph* and *Matrix*. To seamlessly manage the jobs simultaneously submitted to the server by multiple users, asynchronous job scheduling frameworks like *Gearman* and *Supervisor* were implemented. Different JavaScript libraries (including *D3*, *DataTable*, *Crossfilter*, *iCheck*, *gridster* and *DC*) were used at the client side to optimize the user experience through the interactive web-interface, tables and figures. Uploaded data files and programme parameters are stored for future use in a project directory and SQL database, respectively. To ensure data security, CARD uses encrypted data transfer between client and server using Secure Sockets Layer. The complete CARD software code (written in a combination of R, PHP and JavaScript) can be downloaded from the help page on the National Institutes of Health-maintained CARD website (https://card.niaid.nih.gov).

### Data security

The user data are encrypted and securely stored in the CARD database. Only the owner of a project has permission to share data and results with other researchers by specifically inviting them by providing their email addresses. A project can be shared/unshared with any number of researchers. Only the project owner has the authority to execute or alter the analysis parameters. If a project is deleted, it is permanently removed from the database and all the associated files are deleted from the back-end server. To minimize potential security vulnerabilities, the CARD website has been thoroughly tested by the NIAID Office of Cyber Infrastructure and Computational Biology.

### Sample data used for demonstration of CARD functionality

We used three data sets to demonstrate different features of CARD and highlight the utility of the application. We also include template files in ‘CARD format’ in the help page (at https://card.niaid.nih.gov) for new users to format their data for upload. The sample data sets used were as follows.

HDAC-inhibitor screen data: a genome-scale siRNA screen (using a Dharmacon pooled library—pool of 4 unique siRNAs per gene) to identify genes that sensitize cells to HDAC-inhibitor (vorinostat)-induced cell death^17^. This data set also includes an miRNA mimic screen to identify miRNAs contributing to HDAC-inhibitor sensitivity. For each screen, two independent assays were run for cell viability using CTF and for apoptosis using Caspase-Glo 3/7. We label these data sets as HDACi-CTF and HDACi-Casp, respectively. Corresponding transcription-profiling data using RNA-seq were also generated and used for the CARD Gene Expression analysis step. A secondary screen was carried out for 450 genes where the four pooled siRNAs from the primary screen were tested separately. We used the normalized screen data and RNA-seq data from the PubChem (accession numbers 743454, 743458 and 743456) and GEO (accession number GSE56788) databases, respectively.

Parkin screen data: two genome-scale screens to identify genes associated with Parkinson’s disease using a Dharmacon pooled library (four unique siRNAs per gene) and an Ambion single siRNA library (three unique siRNA per gene)^19^. In both screens, Parkin translocation to depolarized mitochondria was measured using high-content microscopy. We used the normalized screen data from the PubChem database (accession numbers 651810 and 651811). The authors did not generate gene expression data directly from the screened cell line. Hence, we used microarray data available for the HeLa cell line from GEO (GSM154355, GSM154356 and GSM154319).

HPV screen data: a genome-scale screen to identify the human genes responsible for mediating repression of the HPV oncogenes E6 and E7 (ref. 28). The screen used a Dharmacon pooled library (four unique siRNAs per gene) and had three biological replicates. A secondary screen was carried out using the four single siRNAs from the primary screen for 511 hit candidates. The authors of this manuscript generously shared the primary and secondary screen data with us. In the absence of available microarray data associated with the screen, we used gene expression data from comparable cell lines in GEO (GSM1186966, GSM1186967 and GSM1186968). Similar to the original paper, we applied CARD analysis only for the positive scores in this screen, as this reports the loss of HPV E2-mediated E6/E7 repression in the screen assay.

### Project creation

Each user needs to create an account using their email address as login ID and a user-defined password. User information is encrypted and securely stored in the CARD database. After account authentication and logging in to the project portal (Supplementary Fig. 1), a project can be created using the ‘+’ icon. Project names can only contain alphanumeric characters (a–z, A–Z and 0–9) and some special characters like ‘_’, ‘.’ and ‘-’ (project names should not contain any space). Each user-defined project must have a unique name. In the project portal, each box in the grid represents a project. The user can drag the boxes to preferred positions and save the customized orientation. On mouse-over, boxes will expand to provide more detailed information on the project, including the ‘Type’ (owned or shared), ‘Owner’ and ‘Shared with’. The user can ‘Share’, ‘Delete’ and ‘Rename’ any project he/she owns. Any project can be easily shared with existing or new users by providing their email addresses. The ‘Manage projects’ link provides a table of all the projects the user has, the list of projects the user has shared with others and the projects that were shared with the user. By clicking the project name, the user can visit the control panel of a specific project. The ‘Help’ link (also available at the top right corner of other pages) provides a list of video tutorials on different functionalities of CARD.

### Input data format

The input data file should be in CARD format, which is a .csv file with the following columns: ‘EntrezID’ or ‘GeneSymbol’ or both, ‘siRNAID’ or ‘shRNAID’ (required only for ‘Single’ siRNA or shRNA, respectively). If ‘siRNA’ library is chosen, ‘Plate’, ‘Well’ and ‘WellAnno’ (annotation of well content type) columns should also be present. The screen data column should be labelled ‘Replicate’ (and if multiple replicates are uploaded, they should be in separate columns with replicate numbers, for example, ‘Replicate1’,’Replicate2’ and so on).

### Genome-wide enrichment of seed sequence

The GESS algorithm, which is also incorporated in the *Off-target Analysis* page, identifies off-targeted genes from seed sequence comparison. Specifically, it attempts to identify transcripts with 3′UTR regions that have a statistically significant enrichment of siRNA seed sequences showing strong phenotypic score in the screen. Seed match frequency for active (SMF_a_) and inactive (SMF_i_) correspond to the fraction of phenotypically active and inactive seed sequences matching the 3′UTR sequence of any given gene. The scatterplot shows the SMF_a_ and SMF_i_ values for all genes, and they are colour-coded based on the *P*-value from GESS (Supplementary Fig. 4). The scatterplot is also interactive, allowing the user to zoom in and mouse-over on each spot to obtain gene details. The figure can also be dragged and zoomed to investigate gene targets of the most active seeds. The table shows results from GESS, where each row corresponds to a gene. In addition to SMF_a_ and SMF_i_, the table also contains ratio (SMF_a_/SMF_i_) and GESS *P*-values before and after corrections (Supplementary Fig. 4). To enable the user to check if potential off-targeted gene also shows an on-target phenotype in the screen, the *Z*-score of the gene-specific siRNA(s) is also included in the ‘Score’ column (NA values indicate genes not targeted by specific siRNA in the screen). A combination of high ratio value and low GESS *P*-value indicates genes that are likely involved in the biological process under study. The user can further filter this table by providing minimum and maximum screen scores.

### Analysis of pooled shRNA screen data using CARD

All the case studies discussed above were from arrayed siRNA screen experiments, where genes were knocked down in one gene per well basis. An alternate RNAi approach uses short hairpin RNA (shRNA) expression vectors, which can be stably integrated into the target cell genome resulting in the stable knockdown of the targeted gene product. In pooled shRNA screens, a target cell population is transfected with a virally expressed shRNA library (usually multiple shRNAs targeting each gene), such that on average, one shRNA from the library is present per cell. Subsequently, shRNA sequences enriched or depleted in cells showing the phenotype of interest are identified by deep sequencing.

To demonstrate the applicability of CARD to such pooled shRNA screens, we downloaded the screen data from Project Achilles^1^ (http://www.broadinstitute.org/achilles), which measured the effect of gene knockdown on viability of 216 cancer cell lines. We used the screen data for MCF7, which is a well-characterized oestrogen receptor positive (ER+) breast cancer cell line. As some of the necessary columns for the analysis of arrayed siRNA screen data (see CARD data analysis workflow below for details) such as PlateID and Well are not relevant for the shRNA pooled screen, these columns are omitted when shRNA screen data set is uploaded to CARD. The first three steps of the CARD analysis, that is, Normalization, Display Data and Off-target analysis are also not applicable for shRNA screen data processing. In the Load Data page, the ‘No Normalization’ option is automatically selected to load the already normalized data. To apply the expression filter, microarray data (with ID GSM803623, GSM803682, and GSM803741) were imported from the GEO database. In the Network Analysis step, we used −2.5, −1.75 and 5 as the threshold for Hits, Non-hits and Minimum Number of Connections with Hits, respectively. In ER+ breast cancer, the Oestrogen Receptor 1 gene (*ESR1*), is the main driver of oncogenesis. As MCF7 is an ER+ breast cancer cell line, the *ESR1* gene is expected to be among the top candidates from the screen data. Although *ESR1* is ranked 1,103 out of 11,939 genes screened based on the screen score, we applied CARD Network Analysis and identified *ESR1* as a central node (hub) in a network of 294 hit genes with direct connections to 62 hit genes (Supplementary Fig. 7).

### Tips to improve data analysis speed

As computationally intense statistical analysis and visualization of genome-scale screen data can be slow, we recommend the following user guidelines. Most of these guidelines are also included in the corresponding analysis pages as mouse-over features on input parameters. During data upload, if the GeneSymbol column is not present, CARD will automatically fetch the gene symbols for the corresponding organism from the Bioconductor resource (https://www.bioconductor.org/), which might take a few extra minutes to complete. If the GeneSymbol column is included, the user should ensure that the organism-specific correct gene symbols are provided. The speed of the CSA algorithm will depend on parameters like ‘Seed Length’ and ‘Minimum Number of Common Seeds’. The number of permutations in RNAiCut determines how fast the process progresses as shown by the progress bar. As some web browsers do not always reload automatically, users are recommended to reload the browser page once any job is complete and to clear their browser cache frequently. The Hit Selection page uses multiple JavaScript libraries, such as D3, Crossfilter and DC, to summarize the data in figures and apply interactive filters. Every time the page is loaded, the client/user’s browser creates the table and figures. If the user applies a filter to one of the figures, the browser needs to recreate all the figures with the set of siRNAs that passes the filter. As genome-scale screen will require processing of megabytes of data on the fly, the response time will depend on the user’s computer specification, browser type and size of the screen data set.

### Caveats to use of external knowledge-bases

RNAiCut, Network and Pathway Analysis steps use external knowledge-bases, such as multiple PPI networks, canonical pathways and gene ontology term characterization. Owing to incompleteness of and potential biases in these resources, the analysis steps using them may inherit those underlying biases. CARD provides the option to select from several leading knowledge-bases for the above analysis steps, however, users should consider the limitations of these resources in the analysis of their screen data.

## Additional information

**How to cite this article:** Dutta, B. *et al*. An interactive web-based application for Comprehensive Analysis of RNAi-screen Data. *Nat. Commun.* 7:10578 doi: 10.1038/ncomms10578 (2016).

## Supplementary Material

Supplementary Information Supplementary Figures 1-7, Supplementary Tables 1-7, and Supplementary References

## Figures and Tables

**Table 1:** Steps in the RNAi screen data analysis workflow and examples of available algorithms and software tools.

Algorithm	Description	Analysis tool(s)
Normalization	Corrects systematic bias present in screen data	CellHTS2 (R bioconductor package)^8^
Common seed analysis	Predicts off-target siRNAs by testing whether siRNAs with shared seed sequence have significantly higher/lower scores compared with the rest	R and Python code^9^
GESS	Identifies likely off-targeted genes	MATLAB code and web-service (http://www.flyrnai.org/gess/)^10^,^11^
RNAiCut	Identifies optimal threshold for hit-selection by integrating screen data with PPI networks	Web-service (does not include any interactive feature) (http://rnaicut.csail.mit.edu)^12^
Expression filter	Flagging and filtering of genes based on their expression status	None
Network analysis	Integration of screen data with PPI data and known biological networks	Cytoscape (a generic desktop application for network analysis and visualization)^16^. Signed PPI (identifies directionality of PPI networks by integrating fly screen data)^15^
Pathway analysis	Identifies biological processes and canonical pathways over-represented in hit genes	GSEA (Java-based desktop application)^14^. DAVID (a generic web-based tool developed for analysing gene expression data)^13^
Integration of results	Combines the results from all the above steps in interactive and user-friendly manner	CARD (https://card.niaid.nih.gov). CARD provides an integrated framework to implement all above steps in the data analysis process

CARD, Comprehensive Analysis of RNAi Data; GESS, Genome-wide Enrichment of Seed Sequence; GSEA, Gene Set Enrichment Analysis; PPI, protein–protein interaction; RNAi, RNA interference; siRNA, short interfering RNA; GSEA, Gene Enrichment Analysis.
