# Corrigendum: An interactive web-based application for Comprehensive Analysis of RNAi-screen Data

**DOI:** 10.1038/ncomms11214

**Published:** 2016-03-21

**Authors:** Bhaskar Dutta, Alaleh Azhir, Louis-Henri Merino, Yongjian Guo, Swetha Revanur, Piyush B. Madhamshettiwar, Ronald N. Germain, Jennifer A. Smith, Kaylene J. Simpson, Scott E. Martin, Eugen Buehler, Iain D. C. Fraser

*Nature Communications* 7: Article number: 10578 10.1038/ncomms10578 (2016); Published 02232016; Updated 03212016

The original version of this article contained an error in the spelling of the author Eugen Buehler, which was incorrectly given as Eugen Beuhler. This has now been corrected in both the PDF and HTML versions of the article.

