# Supplementary material for: An interactive web-based application for Comprehensive Analysis of RNAi-screen Data
Source: Nat Commun. 2016 Feb 23;7:10578. doi: 10.1038/ncomms10578 (PMC5477503; doi:10.1038/ncomms10578)
Supplement: Supplementary Information — Supplementary Figures 1-7, Supplementary Tables 1-7, and Supplementary References [file ncomms10578-s1.pdf]

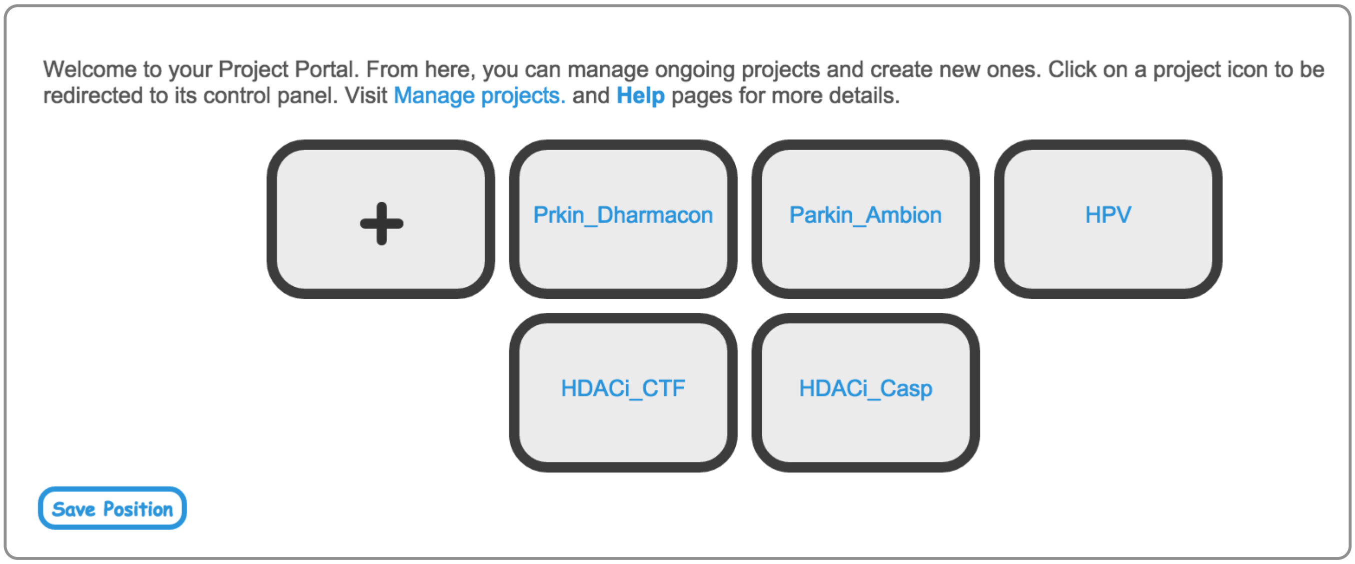

Supplementary Figure 1

CARD *Project Portal* page. Each project is represented by a box in the grid. Details about a project can be obtained by hovering on the corresponding box. The '+' box can be clicked to create new projects.

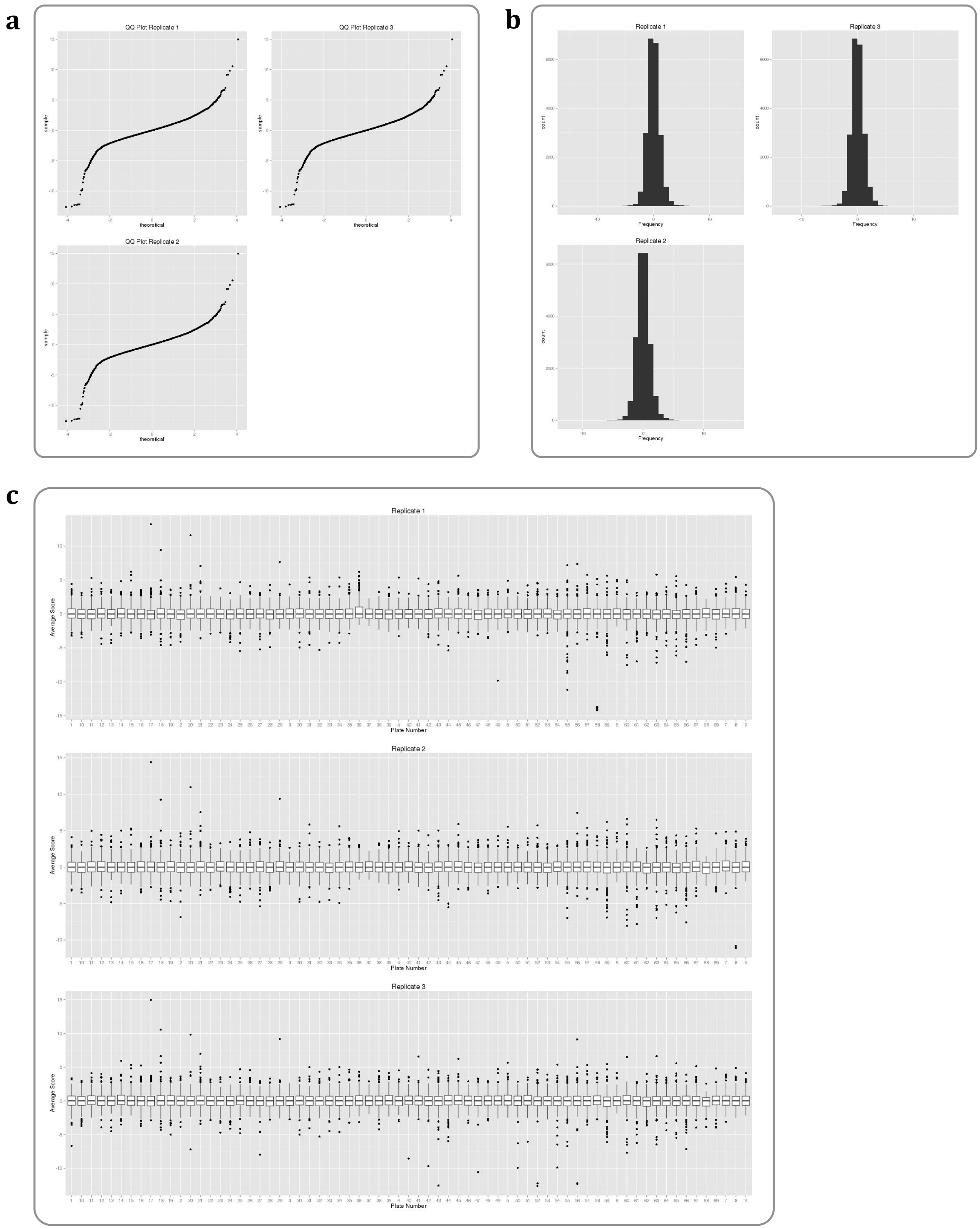

Supplementary Figure 2

Enlarged images of CARD screen data. (a) Quantile-quantile (QQ) plots showing the distribution of screen data with respect to normally distributed data. A straight line implies that the data is normally distributed. (b) Histograms of screen data distribution. (c) Boxplots of the screen data across the plates in a screen.

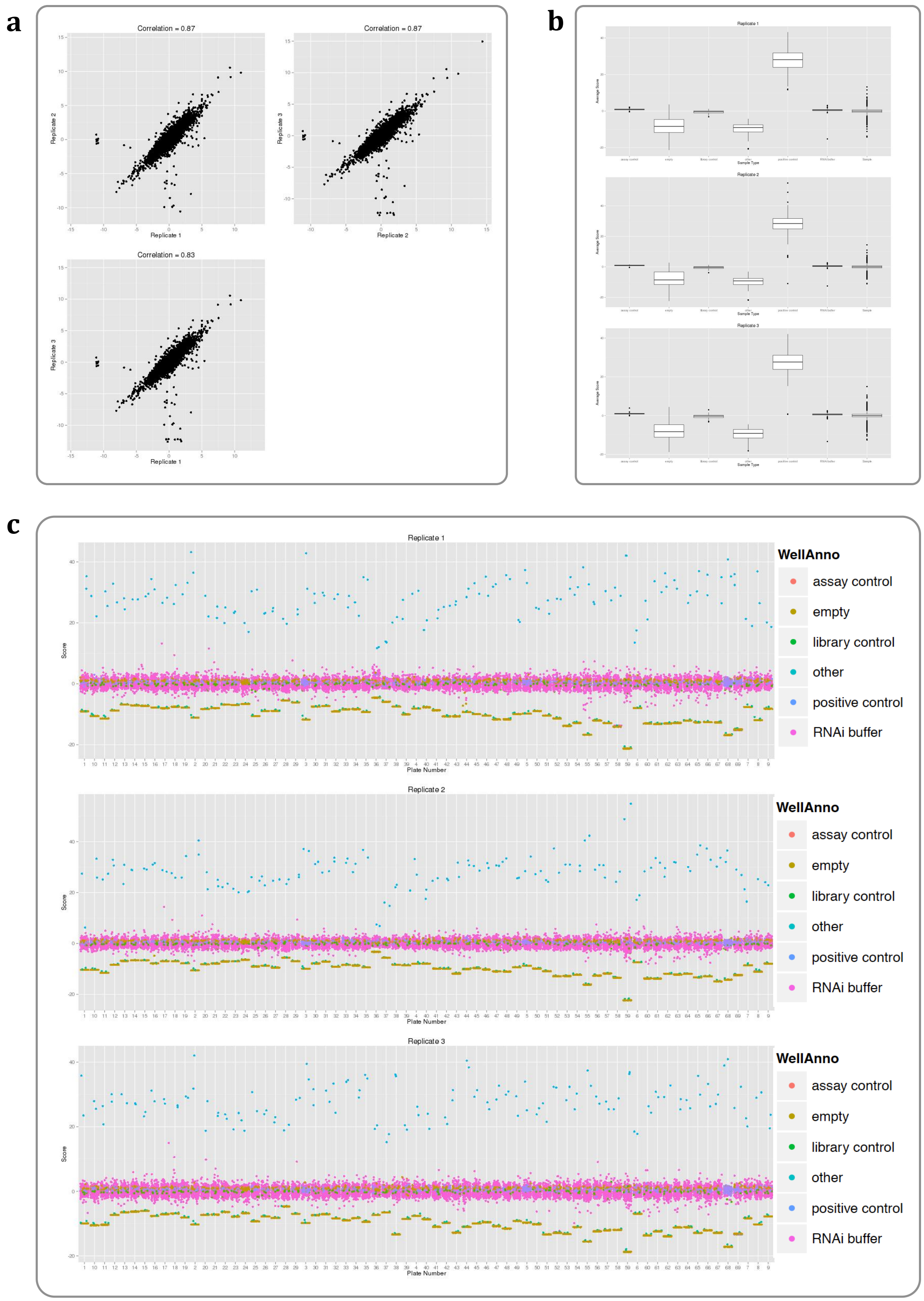

Supplementary Figure 3

Enlarged images of CARD screen data. (a) Pairwise Pearson's correlation of replicate screen scores. (b) Boxplots showing variation of different controls and sample wells pooled from all plates. (c) Jitter-plot of different controls and sample wells across all screen plates.

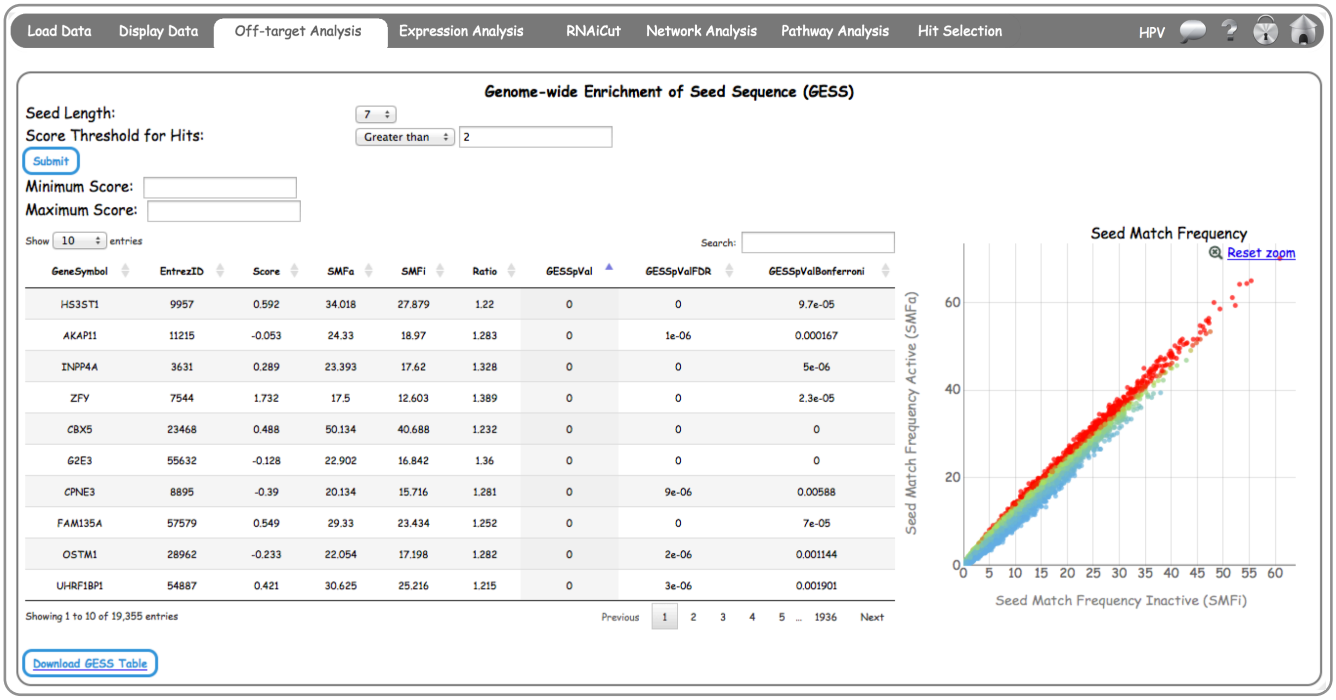

GeneSymbol	EntrezID	Score	SMFa	SMFi	Ratio	GESSpVal	GESSpValFDR	GESSpValBonferroni
HS3ST1	9957	0.592	34.018	27.879	1.22	0	0	9.7e-05
AKAP11	11215	-0.053	24.33	18.97	1.283	0	1e-06	0.000167
INPP4A	3631	0.289	23.393	17.62	1.328	0	0	5e-06
ZFY	7544	1.732	17.5	12.603	1.389	0	0	2.3e-05
CBX5	23468	0.488	50.134	40.688	1.232	0	0	0
G2E3	55632	-0.128	22.902	16.842	1.36	0	0	0
CPNE3	8895	-0.39	20.134	15.716	1.281	0	9e-06	0.00588
FAM135A	57579	0.549	29.33	23.434	1.252	0	0	7e-05
OSTM1	28962	-0.233	22.054	17.198	1.282	0	2e-06	0.001144
UHRF1BP1	54887	0.421	30.625	25.216	1.215	0	3e-06	0.001901

Supplementary Figure 4

CARD *Off-target Analysis* page: Application of the GESS algorithm. Genome-wide enrichment of seed sequence (GESS) identifies putative genes targeted by enriched siRNA seeds in the screen. The results example from the HPV screen dataset are shown as an interactive table and scatterplot. In the scatterplot, genes are presented as circles with colors corresponding to the FDR corrected GESS p-value. Genes targeted by most significantly enriched seeds are highlighted in red.

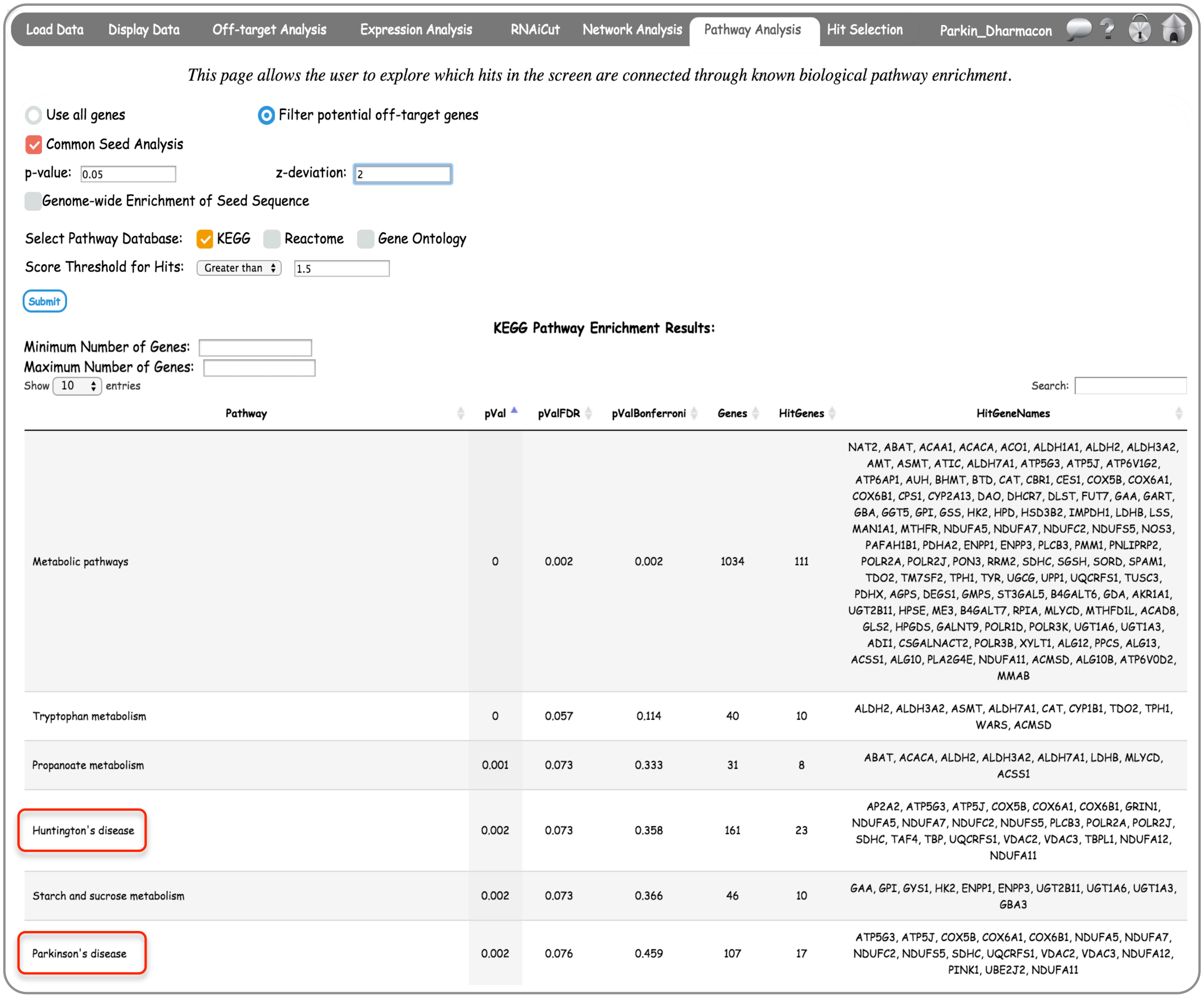

Pathway	pVal	pValFDR	pValBonferroni	Genes	HitGenes	HitGeneNames
Metabolic pathways	0	0.002	0.002	1034	111	NAT2, ABAT, ACAA1, ACACA, ACO1, ALDH1A1, ALDH2, ALDH3A2, AMT, ASMT, ATIC, ALDH7A1, ATP5G3, ATP5J, ATP6V1G2, ATP6AP1, AUH, BHMT, BTD, CAT, CBR1, CES1, COX5B, COX6A1, COX6B1, CPS1, CYP2A13, DAO, DHCR7, DLST, FUT7, GAA, GART, GBA, GGT5, GPI, GSS, HK2, HPD, HSD3B2, IMPDH1, LDHB, LSS, MAN1A1, MTHFR, NDUFA5, NDUFA7, NDUFC2, NDUFS5, NOS3, PAFAH1B1, PDHA2, ENPP1, ENPP3, PLCB3, PMM1, PNLIPRP2, POLR2A, POLR2J, PON3, RRM2, SDHC, SGSH, SORD, SPAM1, TDO2, TM7SF2, TPH1, TYR, UGCG, UPP1, UQCRFS1, TUSC3, PDHX, AGPS, DEGS1, GMPS, ST3GAL5, B4GALT6, GDA, AKR1A1, UGT2B11, HPSE, ME3, B4GALT7, RPIA, MLYCD, MTHFD1L, ACAD8, GLS2, HPGDS, GALNT9, POLR1D, POLR3K, UGT1A6, UGT1A3, ADI1, CSGALNACT2, POLR3B, XYLT1, ALG12, PPCS, ALG13, ACSS1, ALG10, PLA2G4E, NDUFA11, ACMSD, ALG10B, ATP6V0D2, MMAB
Tryptophan metabolism	0	0.057	0.114	40	10	ALDH2, ALDH3A2, ASMT, ALDH7A1, CAT, CYP1B1, TDO2, TPH1, WARS, ACMSD
Propanoate metabolism	0.001	0.073	0.333	31	8	ABAT, ACACA, ALDH2, ALDH3A2, ALDH7A1, LDHB, MLYCD, ACSS1
Huntington's disease	0.002	0.073	0.358	161	23	AP2A2, ATP5G3, ATP5J, COX5B, COX6A1, COX6B1, GRIN1, NDUFA5, NDUFA7, NDUFC2, NDUFS5, PLCB3, POLR2A, POLR2J, SDHC, TAF4, TBP, UQCRFS1, VDAC2, VDAC3, TBPL1, NDUFA12, NDUFA11
Starch and sucrose metabolism	0.002	0.073	0.366	46	10	GAA, GPI, GYS1, HK2, ENPP1, ENPP3, UGT2B11, UGT1A6, UGT1A3, GBA3
Parkinson's disease	0.002	0.076	0.459	107	17	ATP5G3, ATP5J, COX5B, COX6A1, COX6B1, NDUFA5, NDUFA7, NDUFC2, NDUFS5, SDHC, UQCRFS1, VDAC2, VDAC3, NDUFA12, PINK1, UBE2J2, NDUFA11

Supplementary Figure 5

CARD Pathway Analysis page. An example of pathway analysis results are shown using the KEGG database with sample data from the Parkin Dharmacon screen (see Methods). The top enriched pathways from the screen hits are related to metabolic processes and also notably to neurological diseases such as Parkinson's and Huntington's disease.

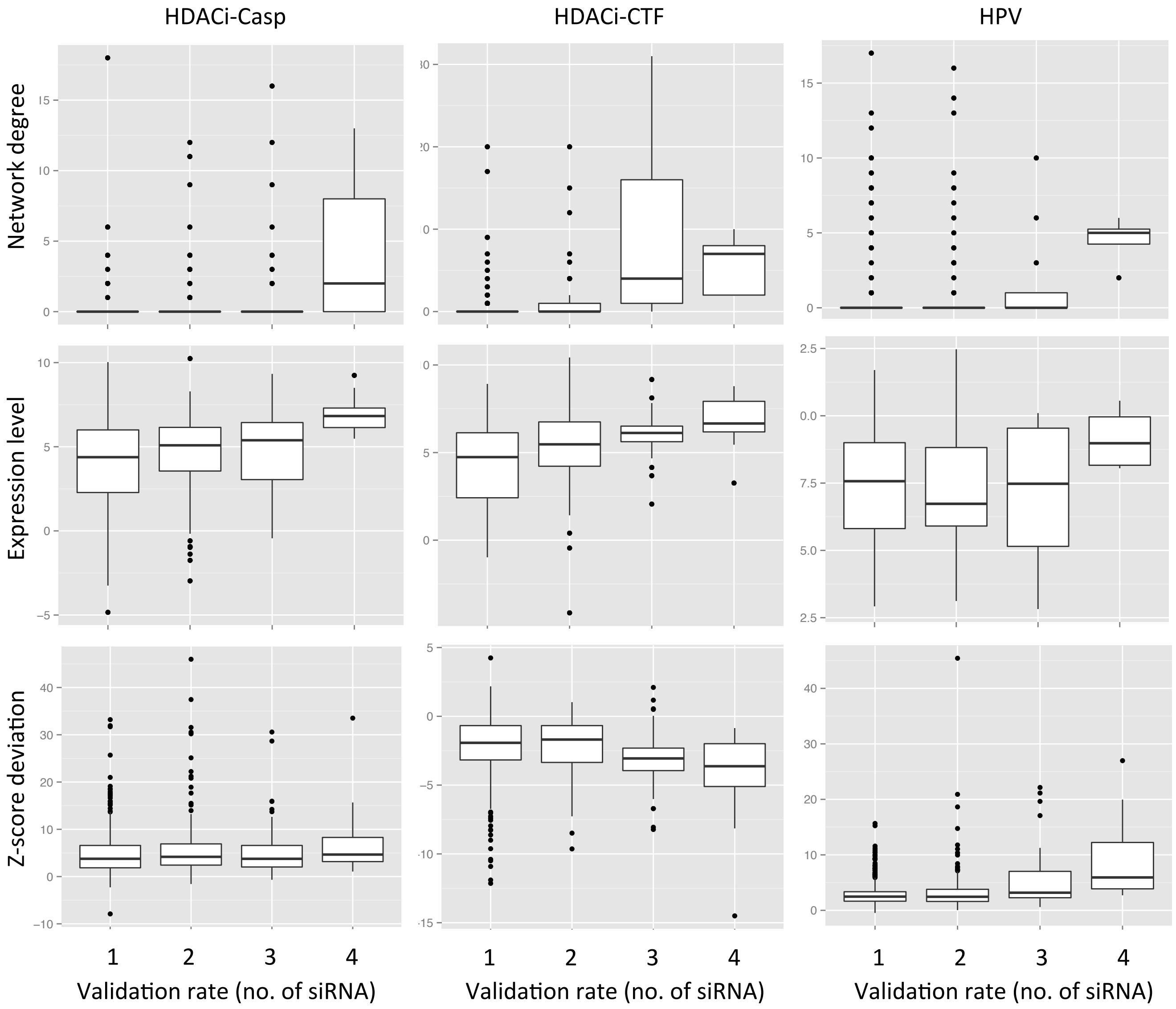

Supplementary Figure 6

CARD analysis metrics show enriched values for validated hits. Different features computed from CARD (Network Degree, Expression Level and Z-score Deviation) are plotted as a function of the number of independent siRNAs (maximum 4) that were validated after deconvolution of the pooled siRNAs in the secondary HDAC inhibitor and HPV screens. Hits not validated by any siRNA are not shown. Genes validated by all 4 siRNAs always show the strongest value for the CARD enrichment metric (note this equates to a lower negative Z-score deviation in the HDACi-CTF screen).

a

Show 10 entries Search:

GeneSymbol	Score	Degree	DegreeFraction	Centrality	EigenVector	ScreenHit
SUMO2	-1.984	74	0.061	397423194248.914	1	No
ESR1	-1.9225	62	0.076	713316104393.376	0.838	No
CSNK1A1	-2.5545	53	0.21	707156277166.875	0.716	Yes
UBL4A	-3.099	53	0.203	700426998832.239	0.716	Yes
RPS3	-3.576	52	0.268	1220548199126.92	0.703	Yes
RPS4X	-4.687	47	0.272	1155378467528.89	0.635	Yes
RPS6	-3.656	47	0.261	1116596530986.26	0.635	Yes
RPS16	-2.501	46	0.299	1111146739787.63	0.622	Yes
RPS3A	-3.666	46	0.26	1063739957304.05	0.622	Yes
RPS13	-4.736	45	0.298	1011495581299.36	0.608	Yes

Showing 1 to 10 of 294 entries Previous 1 2 3 4 5 ... 30 Next

b

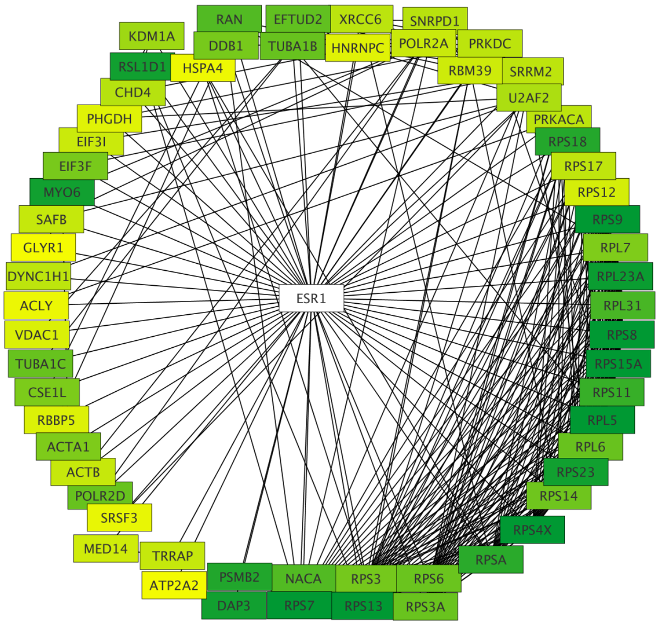

Supplementary Figure 7

Analysis of shRNA screen data in CARD. Pooled shRNA screen coupled with next generation sequencing data (obtained from Project Achilles) from the MCF7 cell line was analyzed using CARD. The network analysis was carried out with both hit and non-hit genes (Supplementary Methods). As the MCF7 cell line is classified as a ER+ breast cancer subtype, the ESR1 gene plays an important role. (a) Although the score of the *ESR1* gene was below threshold for hit selection (ranked 1103 out of 11939 genes screened), the CARD network analysis shows it has the second largest Eigenvector and Degree (highlighted in red). Hence, the *ESR1* gene plays a key role in connecting the hit genes from the screen data. (b) a network module connecting *ESR1* gene with other hit genes from the screen. The network was generated by Cytoscape from Cytoscape compatible input files (.gml) output from CARD.

Supplementary Table 1

CARD format file for screen with individual siRNA.

siRNAID	EntrezID	WellAnno	GeneSymbol	PlateID	Row	Column	Replicate1
s816	1	Sample	A1BG	QDA102104	1	1	0.89
s817	1	Sample	A1BG	QDA103282	1	1	-1.35
s818	1	Sample	A1BG	QDA103382	1	1	-2.44
s819	2	Sample	A2M	QDA102214	1	1	-1.99
s820	2	Sample	A2M	QDA102407	1	1	0.42
s821	2	Sample	A2M	QDA102483	1	1	-0.35
s822	9	Sample	NAT1	QDA102407	1	3	0.05
s823	9	Sample	NAT1	QDA102483	1	3	-0.05
s824	9	Sample	NAT1	QDA102214	1	3	0.09
s825	10	Sample	NAT2	QDA102407	1	5	0.54
s826	10	Sample	NAT2	QDA102483	1	5	-2.84
s827	10	Sample	NAT2	QDA102214	1	5	-0.26
s828	12	Sample	SERPINA3	QDA102214	1	7	0.03
s829	12	Sample	SERPINA3	QDA102407	1	7	-0.02
s830	12	Sample	SERPINA3	QDA102483	1	7	-1.11

Example of required fields in ‘CARD format’ screen data file for a screen with individual siRNAs (siRNA_ID field is required). Plate position can be denoted either by ‘Well’ field with alpha numeric plate position, or by separate ‘Row’ and ‘Column’ fields. GeneSymbol is optional as EntrezID is used as the primary gene identifier.

Supplementary Table 2

CARD format file for screen with siRNA pools.

Plate	Well	WellAnno	GeneSymbol	EntrezID	Replicate1
52	G15	Sample	A1BG	1	0.23
2	G19	Sample	A2M	2	-0.15
7	D19	Sample	NAT1	9	0.98
14	N10	Sample	NAT2	10	1.61
25	I13	Sample	SERPINA3	12	1.41
12	I09	Sample	AADAC	13	1.36
48	N06	Sample	AAMP	14	1.05
15	C21	Sample	AANAT	15	0.94
2	F05	Sample	AARS	16	2.20
16	N03	Sample	ABAT	18	1.74
2	M04	Sample	ABCA1	19	1.84
5	G11	Sample	ABCA2	20	1.76
11	B20	Sample	ABCA3	21	1.28
10	F11	Sample	ABCB7	22	0.73
17	I18	Sample	ABCF1	23	0.18
5	G17	Sample	ABCA4	24	0.04
19	D16	Sample	ABL1	25	-1.30
27	H03	Sample	ABP1	26	1.36
21	H10	Sample	ABL2	27	-0.43

Example of required fields in 'CARD format' screen data file for a screen with pooled siRNAs (siRNA_ID field is not required). Plate position can be denoted either by 'Well' field with alpha numeric plate position, or by separate 'Row' and 'Column' fields. GeneSymbol is optional as EntrezID is used as the primary gene identifier.

Supplementary Table 3

CARD format file for siRNA sequence upload.

siRNAID	EntrezID	GeneSymbol	Sequence
s1043	117	ADCYAP1R1	UAACGGUUCACCUUCCAGCtt
s1044	117	ADCYAP1R1	ACAAGGAUGACGAUAAUGCca
s1045	117	ADCYAP1R1	UAGUGGAUUCCGAAUAGUGgg
s1055	123	ADFP	UGAGAGGUAGAGCUUAUCCtg
s1056	123	ADFP	UACUGUUCUACCAACAGCUct
s1057	123	ADFP	UGCUUAGCUUCUUUAACCCtg

Example of required fields in 'CARD format' siRNA sequence data file. Note that CARD will take only the first 19 nucleotides of siRNA sequence, so data files that include two base overhangs at the end of the siRNA sequence (as shown in the 'Sequence' column) are acceptable for upload. Either 'sense' or 'antisense' sequence can be used, but the user must check the appropriate radio button to choose one or the other on the CARD *Off-target Analysis* page.

Supplementary Table 4

CARD format file for mRNA expression data upload.

EntrezID	Gene	Value	ABS_CALL
16728	L1cam	6.949941072	Present
13798	En1	6.882419292	Absent
235281	Scn3b	6.730827413	Absent
11513	Adcy7	9.094310768	Present
80879	Slc16a3	6.97746998	Present
242773	Slc45a1	6.813702963	Absent
258502	Olfr279	6.761853037	Absent
20737	Spn	7.058786204	Present
52915	Zmiz2	9.042783851	Present
66313	Smurf2	7.455488333	Present
237831	Slc13a5	6.892523388	Absent

Example of required fields in an mRNA expression data file. The file must contain columns with headers EntrezID and Value (expression levels). The file may also contain Present/Absent calls (ABS_CALL) and/or detection p-values (DetectionPval). If multiple replicates are present, columns containing expression levels should have headers with Value appended by the replicate number.

Supplementary Table 5

CARD parameters used in analysis of HDACi and HPV siRNA screens.

Screen Name	Property	Value
**HDACi-Casp primary screen**	Z-score*	> 5.13
	Network degree	> 0
	Expression level	> 3
	CSA p-value	> 0.05
	Z-score deviation	> 5.13
**HDACi-CTF primary screen**	Z-score*	< -2.91
	Network degree	> 0
	Expression level	> 3
	CSA p-value	> 0.05
	Z-score deviation	< -2.91
**HPV primary screen**	Z-score*	> 2
	Network degree	> 0
	Expression level	> 5
	CSA p-value	> 0.05
	Z-score deviation	> 2
**HDACi-Casp secondary screen**	Z-score*	> 2.2
**HDACi-CTF secondary screen**	Z-score*	< 0.6
**HPV secondary screen**	Z-score*	> 2

The list of properties and their corresponding cutoffs used for hit-selection in CARD. Asterisks indicate the z-score cutoffs used in the original studies.

Supplementary Table 6

The list of parameters used for analyzing the Parkin screen datasets in CARD.

Property	Parkin-Dharmacon	Parkin-Ambion
Z-score	> 2	> 1.5
Expression status	Present or Missing	Present or Missing
CSA p-value	> 0.05	> 0.05
Z-score deviation	> 2	> 1.5
Minimum number of siRNAs	Not applicable	>=2
Network Degree	> 0	> 0
Non-hit threshold for network analysis	> 1	> 0.75

Supplementary Table 7

Identification of likely screen hits in CARD through Network Analysis.

Gene	Reference
CTNNB1	Identification of Pivotal Cellular Factors Involved in HPV-Induced Dysplastic and Neoplastic Cervical Pathologies[2].
TRRAP	SMCX and components of the TIP60 complex contribute to E2 regulation of the HPV E6/E7 promoter[3].
KAT5	Destabilization of TIP60 by human papillomavirus E6 results in attenuation of TIP60-dependent transcriptional regulation and apoptotic pathway[4].<br>SMCX and components of the TIP60 complex contribute to E2 regulation of the HPV E6/E7 promoter[3].
CREBBP	The human papillomavirus type 16 E6 oncoprotein can down-regulate p53 activity by targeting the transcriptional coactivator CBP/p300[5].<br>CBP/p300 in cell growth, transformation, and development[6].
PCNA	Increased expression of cellular RNA-binding proteins in HPV-induced neoplasia and cervical cancer[7].<br>Expression of PCNA is associated with the presence of HPV DNA in skin warts[8].

The list of genes with moderate scores in the HPV screen (>1.5 and < 2), found to be most connected (≥7 connections) with screen hits (defined by screen score >2) using Network Analysis in Supplementary Fig. 12. All of the genes are found to be linked with HPV-associated disease based on existing literature.

Supplementary References

1 Cowley, G. S. *et al.* Parallel genome-scale loss of function screens in 216 cancer cell lines for the identification of context-specific genetic dependencies. *Scientific Data* **1**, 140035, doi:10.1038/sdata.2014.35 (2014).

2 Mattarocci, S. *et al.* Identification of pivotal cellular factors involved in HPV-induced dysplastic and neoplastic cervical pathologies. *Journal of cellular physiology* **229**, 463-470, doi:10.1002/jcp.24465 (2014).

3 Smith, J. A. *et al.* SMCX and components of the TIP60 complex contribute to E2 regulation of the HPV E6/E7 promoter. *Virology* **468-470**, 311-321, doi:10.1016/j.virol.2014.08.022 (2014).

4 Jha, S. *et al.* Destabilization of TIP60 by human papillomavirus E6 results in attenuation of TIP60-dependent transcriptional regulation and apoptotic pathway. *Molecular cell* **38**, 700-711, doi:10.1016/j.molcel.2010.05.020 (2010).

5 Zimmermann, H., Degenkolbe, R., Bernard, H. U. & O'Connor, M. J. The human papillomavirus type 16 E6 oncoprotein can down-regulate p53 activity by targeting the transcriptional coactivator CBP/p300. *J Virol* **73**, 6209-6219 (1999).

6 Goodman, R. H. & Smolik, S. CBP/p300 in cell growth, transformation, and development. *Genes & development* **14**, 1553-1577 (2000).

7 Fay, J., Kelehan, P., Lambkin, H. & Schwartz, S. Increased expression of cellular RNA-binding proteins in HPV-induced neoplasia and cervical cancer. *Journal of medical virology* **81**, 897-907, doi:10.1002/jmv.21406 (2009).

8 Lu, S., Syrjanen, K., Havu, V. K. & Syrjanen, S. Expression of PCNA is associated with the presence of HPV DNA in skin warts. *Archives of dermatological research* **289**, 35-39 (1996).
